# Discovery of dominant and dormant genes from expression data using a novel generalization of SNR for multi-class problems

**DOI:** 10.1186/1471-2105-9-425

**Published:** 2008-10-09

**Authors:** Yu-Shuen Tsai, Chin-Teng Lin, George C Tseng, I-Fang Chung, Nikhil Ranjan Pal

**Affiliations:** 1 Institute of Biomedical Informatics, National Yang-Ming University, Taipei, Taiwan; 2 Department of Electrical and Control Engineering, Department of Computer Science, Brain Research Center, National Chiao-Tung University, Hsinchu, Taiwan; 3 Department of Biostatistics, Department of Human Genetics, Department of Computational Biology, University of Pittsburgh, USA; 4 Electronics and Communication Sciences Unit, Indian Statistical Institute, Calcutta, India

## Abstract

**Background:**

The Signal-to-Noise-Ratio (SNR) is often used for identification of biomarkers for two-class problems and no formal and useful generalization of SNR is available for multiclass problems. We propose innovative generalizations of SNR for multiclass cancer discrimination through introduction of two indices, Gene Dominant Index and Gene Dormant Index (GDIs). These two indices lead to the concepts of dominant and dormant genes with biological significance. We use these indices to develop methodologies for discovery of dominant and dormant biomarkers with interesting biological significance. The dominancy and dormancy of the identified biomarkers and their excellent discriminating power are also demonstrated pictorially using the scatterplot of individual gene and 2-D Sammon's projection of the selected set of genes. Using information from the literature we have shown that the GDI based method can identify dominant and dormant genes that play significant roles in cancer biology. These biomarkers are also used to design diagnostic prediction systems.

**Results and discussion:**

To evaluate the effectiveness of the GDIs, we have used four multiclass cancer data sets (Small Round Blue Cell Tumors, Leukemia, Central Nervous System Tumors, and Lung Cancer). For each data set we demonstrate that the new indices can find biologically meaningful genes that can act as biomarkers. We then use six machine learning tools, Nearest Neighbor Classifier (NNC), Nearest Mean Classifier (NMC), Support Vector Machine (SVM) classifier with linear kernel, and SVM classifier with Gaussian kernel, where both SVMs are used in conjunction with one-vs-all (OVA) and one-vs-one (OVO) strategies. We found GDIs to be very effective in identifying biomarkers with strong class specific signatures. With all six tools and for all data sets we could achieve better or comparable prediction accuracies usually with fewer marker genes than results reported in the literature using the same computational protocols. The dominant genes are usually easy to find while good dormant genes may not always be available as dormant genes require stronger constraints to be satisfied; but when they are available, they can be used for authentication of diagnosis.

**Conclusion:**

Since GDI based schemes can find a small set of dominant/dormant biomarkers that is adequate to design diagnostic prediction systems, it opens up the possibility of using real-time qPCR assays or antibody based methods such as ELISA for an easy and low cost diagnosis of diseases. The dominant and dormant genes found by GDIs can be used in different ways to design more reliable diagnostic prediction systems.

## Background

Many studies have investigated the mechanism of carcinogenesis by analyzing the gene expression profiles from microarray data. Accurate diagnosis of different categories of cancers or identification of subgroups with homogeneous molecular signature is important for proper treatment and prognosis. The application of gene expression data for these tasks is challenging because of its high dimensional nature and the noisy characteristics. Since the number of genes contained in each chip is far exceeding the number of available samples, the standard statistical methods for classification often do not work well. Therefore, identification of informative genes related to a set of diseases is an important subject in the field of biomedical informatics at least for two reasons: understanding the roles played by the genes in cancer biology and development of tools for efficient and accurate diagnostic prediction.

Many novel classification, clustering and prediction methodologies have been suggested to analyze gene expression data [1-4]. Here we need to deal with two problems: identification of marker genes (this is a problem of dimensionality reduction) and use of the marker genes for designing a diagnostic prediction system. For the second problem many machine learning tools, such as Neural Networks, Decision Trees, Nearest Neighbor Classifier, Naive Bayes classifier, Support Vector Machines have been used [5-8]. For the problem of gene selection also many methods have been proposed [2,4,7-10]. Gene selection methods can further be grouped into two categories: *linear *methods and *non-linear *methods.

The linear methods are very intuitive which exploit the linear relation between expression levels and the status of the disease. In other words, for a two-class problem, say Acute Lymphoblastic Leukemia (ALL) and Acute Myelogenous Leukemia (AML), the high expression level may correspond to ALL while a low expression level may correspond to AML or vice versa. Two such indices are Signal-to-Noise ratio (SNR) [2] and correlation [7]. The SNR for a gene *g *is defined as SNR(*g*) = (*μ*_1_(*g*) - *μ*_2_(*g*))/(*σ*_1_(*g*) + *σ*_2_(*g*)), where *μ*_*i*_(*g*) and *σ*_*i*_(*g*) are the mean and standard deviation of expression levels of a gene *g *for samples in class *i *(*i *= 1, 2), respectively. The authors in [7] adopted several formulae (Euclidean distance, Pearson correlation, SNR, etc.) for measuring the similarity between the expression levels of a gene *g *and an ideal gene **g**_*ideal *_in a 2-class problem, where an ideal gene pattern was defined by **g**_*ideal *_= (*g*_*ideal*,1_, ⋯, *g*_*ideal*,*G*_), *g*_*ideal*,*j *_= 1, if the *j*th sample is from class 1, otherwise *g*_*ideal*,*j *_= 0; ∀ *j *= 1, ⋯, *G*. The ideal values can also be taken as 0 for class 1 and 1 for the class 2. Let **x**_*g *_be the vector consisting of the expression values for a gene *g *for all samples. Now the Pearson correlation or cosine similarity between the two vectors **g**_*ideal *_and **x**_*g *_can be used to rank the genes. Although very intuitive, these methods are neither easy to generalize to multiclass, nor such methods can take into account non-linear interaction between genes. The BW ratio [4] is a linear index that can be used for multiclass problems, but it is less intuitive and it is not easy to visualize its behavior.

Note that, there have been attempts to adapt two-class methods such as correlation for multiclass problems using the one-vs-all strategy [11]. In [11], first a set of genes is selected based on ANOVA. Then using this short-listed genes, a set of important genes is identified for each class by casting the problem into a two class problem. We call these method as ANOVA+Correlation method. For example, in a *k*-class problem, to get a set of important genes for class *c*, samples from class *c *are considered from class 1 and all samples from the remaining classes pooled together are treated as class 2. Then the correlation, as explained, in the previous paragraph is computed. Such a method will select strong marker genes, but may also select poor ones because the pooled class will have a much stronger and undesirable effect on the correlation than the class under consideration. Similarly, using the OVA strategy the SNR can also be used to select genes for a multiclass problem [12]. We shall call this method as OVA.SNR. In the OVA.SNR approach, for a *k*-class problem, to select useful genes, say, for class 1, the data set is divided into two groups, data from class 1 and data from the the remaining 2 to *k *classes. Although such methods may find useful genes, in this case, the mean and standard deviation of the pooled group may not (usually will not) represent any useful information about the remaining classes. For example, in a 3-class problem, suppose for a gene, samples from each of the three classes are normally distributed (this is an assumption made while using ANOVA type tests). For simplicity, suppose we have *n *samples from each of the three classes and the mean and standard deviation computed from these samples for the three classes are *μ*_*i*_, *σ*_*i*_; *i *= 1, ⋯, 3, respectively. In the OVA.SNR approach, the mean of the second group, $\mu =\frac{\mu_{2}+\mu_{3}}{2}$ does not represent the central tendency of the pooled group and hence it does not represent any useful information about the structure of the remaining two classes. Moreover, when samples from class 2 and class 3 are normally distributed with two different means, the pooled samples will not be normally distributed. Hence, OVA schemes, which use mean of the pooled class, for gene selection is not conceptually appealing, although such approaches may find useful discriminatory genes.

On the other hand, the non-linear methods can take into account non-linear interaction between genes. There are several such methods, for example, online feature selection using neural network [10], SVM-based recursive feature elimination (SVM-RFE) [9], and the maximum margin criterion-based recursive feature elimination (MMC-RFE) [8]. In [10], the authors have considered the non-linear interaction between genes as well as that between genes and the tool used for gene selection. Although in [10] they have successfully discovered a small set of biomarkers for accurate prediction of cancer subgroups, the behavior of non-linearly interacting genes is less interpretable than the linearly interacting genes for making simple decision rules. The SVM-RFE is a quite popular method of feature selection in an iterative manner. This method makes use of repeated training of a SVM classifier with a progressively reduced set of features. In every iteration, some of the less important features are removed. For a two-class problem, the SVM classifier finds the weight vector, **w **∈ *R*^*p*^, *p *is the number of genes, associated with the hyperplane that maximizes the margin of separation. The SVM-REF algorithm, trains SVM with all available genes first and finds the optimal weight vector **w **∈ *R*^*p*^. Then it computes a Ranking Criterion, RC, for each gene. A possible choice of RC is (*w*_*i*_)^2^. Then either a single gene (or a set of genes) with the smallest values of RC is removed and the process is then repeated with the reduced set of genes.

Here we aim to develop a gene selection method which is intuitive, can find useful marker genes and can be viewed as a true generalization of SNR. The GDI is akin to the SNR, which is widely used in two-class gene selection problems [2], but GDI can be applied to *multicategory *problems, and identifies dominant and dormant genes. We define two indices named, *Gene Dominant Index *(*GDI*_*Dom*_) and *Gene Dormant Index *(*GDI*_*Dor*_). The *GDI*_*Dom *_leads to the novel concept of *Dominant Genes *while the other index leads to the concept of *Dormant Genes*. A dominant gene is over-expressed in only one of the classes and under-expressed in the remaining classes, and thus has a very strong class specific signature. A dormant gene, on the other hand, is *under*-expressed in only one of the classes but *over*-expressed in the remaining classes, and thus also has a strong class specific signature. Clearly, dominant or dormant genes are good biomarkers, if they exist, and they are likely to play key roles in identifying sub-types/classes of disease. In order to reduce the effect of the finite sample size, we randomly select a part of the data to find a list of dominant and dormant genes. This process of random partition of data and computation of GDIs are repeated 100 times. The frequency with which different genes appear in the list of dominant and dormant genes is then computed. Since really good dominant and dormant genes are expected to appear more frequently, we select a set of most frequently occurring dominant (dormant) genes. A set of strong dominant and/or dormant genes, thus selected, can be used to design reliable diagnostic systems. Further details about the definitions and procedures can be found in the Materials and Methods section.

We want to emphasize that many genes may have discriminating power and hence can be considered marker genes but the dominant and dormant genes are special types of markers. Thus dominant and dormant genes are markers genes but all marker genes are not necessarily dominant/dormant genes and GDI is designed to identify dominant/dormant genes, if present. However, even if there are not many good dominant/dormant genes and we select a set of markers based on GDIs, such a set will do a good job of classification.

To compare the performance of our methods, we shall use six classifiers for diagnostic prediction: NMC, NNC, SVM with linear kernel, and SVM with Gaussian kernel. Each of the two SVMs is realized using both the OVA and OVO strategies and this makes the total number of classifiers to six. Our method is tested on four multi-class cancer data sets. We shall see later that our proposed methods can find a small set of discriminating biomarkers with excellent prediction accuracy.

## Results and discussion

Four multicategory microarray gene expression data sets, namely, SRBCT (Small Round Blue Cell Tumors) [13], Leukemia [14], CNS (Central Nervous System Tumors) [15], and Lung Cancer [16] are used in this study for detailed analysis. We divide our discussion into three subsections, the biological relevance of some of the dominant/dormant genes, visual assessment of the dominant/dormant marker genes, and comparison of classifier performance. The results obtained using SRBCT, Leukemia, and CNS are compared with those in [8]. The Lung Cancer data set (not used in [8]) is further used to show the effectiveness of our method. Details of the data sets can be found in Materials and Methods. We have followed the same experimental protocols as in [8] to make a proper comparison. Additionally, we have implemented the multiclass version correlation based method (ANOVA+Correlation) and SNR (OVA.SNR) for comparison of performance.

### Biological relevance of some dominant/dormant genes

Tables 1, 2, 3, 4, obtained by the Algorithm *Gene Selection *(see Materials and Methods), list the sets of dominant and dormant genes for the SRBCT, Leukemia, CNS, and Lung Cancer data sets, respectively. In Table 1 for the SRBCT data set, four of the most dominant genes, one for each class, identified by the *GDI*_*Dom*_are (a) FCGRT, (b) WAS, (c) AF1Q, (d) FGFR4. The gene FCGRT (Fc fragment of IgG, receptor) has an EWS (Ewing sarcomas) specific signature because it is moderate to highly upregulated for the EWS group and is downregulated for the other three groups. This gene is known to play significant roles in other types of cancers too. For example, in [17] authors suggested a set of 26 prognostic genes that can provide predictive information on the survival of patients suffering from lung cancer. They found that a higher expression level of FCGRT relates to a better survival outcome.

**Table 1:** Details of the selected genes by the frequency-based method for the SRBCT data set

	Class	Image ID	Gene Symbol	Frequency	Ave. GDI	*p*-value	*q*-value
**Dom**	**EWS**	770394	FCGRT	100	1.88	0	0
		814260	FVT1	100	1.43	0	0
		377461	CAV1	99	1.46	0	0
		1435862	CD99	94	1.37	0	0
		866702	PTPN13	88	1.28	0	0
	
	**BL**	236282	WAS	100	2.19	0	0
		183337	HLA-DMA	67	1.82	0	0
		745019	EHD1	51	2.03	0	0
		1469292	PIM2	24	1.90	0	0
		47475	CYFIP2	24	1.85	0	0
	
	**NB**	812105	AF1Q	99	1.65	0	0
		134748	GCSH	64	1.45	0	0
		756401	RHEB	56	1.41	0	0
		325182	CDH2	33	1.38	0	0
		629896	MAP1B	32	1.32	0	0
	
	**RMS**	784224	FGFR4	100	1.60	0	0
		796258	SGCA	96	1.27	0	0
		244618	FNDC5	65	1.18	0	0
		839552	NCOA1	42	1.14	2.60E-06	0.0002
		769716	NF2	38	1.12	2.60E-06	0.0001

**Dor**	**EWS**	295985	CDK6	100	1.37	0	0
		448386	PBX3	73	0.96	5.11E-05	0.0011
		842820	PABPC4	43	0.78	0.0011	0.0115
		214572	CDK6	39	0.77	0.0003	0.0039
		366009	LYAR	24	0.93	0.0078	0.0457
	
	**BL**	204545	ANTXR1	70	2.04	0	0
		154472	FGFR1	68	2.15	0	0
		66552	C20orf194	57	2.12	0	0
		345538	CTSL	50	2.27	0	0
		142788	SERPINH1	21	2.04	0	0
	
	**NB**	810057	CSDA	85	1.29	0	0
		753418	VASP	62	1.16	1.73E-06	8.16E-05
		686164	DGKZ	42	1.13	6.07E-06	0.0002
		769716	NF2	34	1.12	9.53E-06	0.0003
		128126	CD55	33	1.47	0.0003	0.0038
	
	**RMS**	897177	PGAM1	73	0.75	0.0004	0.0053
		295986	EBP	61	0.80	0.0004	0.0053
		711959	POLR3C	41	0.72	0.0016	0.0150
		163174	TCEA1	31	0.76	0.0016	0.0148
		306921	EEF1E1	23	0.72	0.0028	0.0224

Using the training data, for each class the top five most frequently occurring genes are selected.

**Table 2:** Details of the selected genes by the frequency-based method for the Leukemia data set

	Class	Probe ID	Gene Symbol	Frequency	Ave. GDI	*p*-value	*q*-value
**Dom**	**ALL**	1389_at	MME	96	1.98	0	0
		32847_at	MYLK	62	2.02	0	0
		32872_at	ESTs	58	1.73	0	0
		35164_at	WFS1	52	2.05	0	0
		37280_at	SMAD1	25	1.87	0	0
	
	**MLL**	34306_at	MBNL1	99	1.43	0	0
		40763_at	MEIS1	92	1.41	0	0
		36777_at	KLRK1	83	1.42	0	0
		1065_at	FLT3	56	1.20	0	0
		34583_at	FLT3	30	1.30	0	0
	
	**AML**	39566_at	CHRFAM7A	46	1.89	0	0
		41752_at	GHITM	39	1.51	0	0
		38710_at	OTUB1	31	1.54	0	0
		37187_at	CXCL2	22	1.53	0	0
		36162_at	BSG	21	1.47	0	0

**Dor**	**ALL**	33412_at	LGALS1	94	1.66	0	0
		37403_at	ANXA1	90	1.74	0	0
		37809_at	HOXA9	62	1.59	0	0
		41448_at	HOXA10	54	1.64	0	0
		31575_f_at	ESTs	33	1.65	0	0
	
	**MLL**	1674_at	YES1	69	1.03	0	0
		1325_at	SMAD1	43	0.97	9.54E-07	2.80E-05
		539_at	RYK	39	1.05	0	0
		1971_g_at	FHIT	33	0.90	9.54E-07	3.02E-05
		37527_at	ELK3	28	0.98	9.54E-07	2.81E-05
	
	**AML**	41747_s_at	MEF2A	50	1.89	0	0
		41503_at	ZHX2	49	1.88	0	0
		37988_at	CD79B	37	1.98	0	0
		37710_at	MEF2C	34	2.04	0	0
		40966_at	STK39	32	2.12	0	0

Using the training data, for each class the top five most frequently occurring genes are selected.

**Table 3:** Details of the selected genes by the frequency-based method for the CNS data set

	Class	Probe ID	Gene Symbol	Frequency	Ave. GDI	*p*-value	*q*-value
**Dom**	**MD**	M93119_at	INSM1	79	1.65	8.98E-06	0.0018
		HG884-HT884_s_at	ESTs	32	1.42	0.0006	0.0224
		S82240_at	RND3	29	1.49	0.0002	0.0116
		Y09836_at	MAP1B	25	1.35	0.0006	0.0225
		D80004_at	KIAA0182	22	1.34	0.0009	0.0263
	
	**MGlio**	M93426_at	PTPRZ1	55	1.93	9.82E-06	0.0019
		X03100_cds2_at	HLA-DPA1	47	1.69	0.0006	0.0223
		X86693_at	SPARCL1	43	1.44	5.21E-05	0.0046
		D38522_at	SYT11	35	1.98	2.67E-05	0.0033
		U55258_at	ESTs	26	1.43	0.0010	0.0270
	
	**Rhab**	D84454_at	SLC35A2	72	2.11	1.68E-06	0.0007
		D17400_at	PTS	38	1.77	1.21E-05	0.0021
		U47621_at	SC65	25	1.72	1.71E-05	0.0028
		L38969_at	THBS3	23	1.95	2.11E-05	0.0030
		D30755_at	TNIP1	21	1.82	2.71E-05	0.0033
	
	**Ncer**	U92457_s_at	GRM4	66	4.30	0	0
		X63578_rna1_at	PVALB	64	3.66	0	0
		U79288_at	KIAA0513	62	3.38	0	0
		HG2259-HT2348_s_at	ESTs	32	4.62	2.81E-07	0.0003
		D26070_at	ITPR1	30	3.93	0	0
	
	**PNET**	K02882_cds1_s_at	IGHD	55	1.15	0.0002	0.0117
		X14830_at	CHRNB1	29	1.21	0.0012	0.0307
		M80397_s_at	POLD1	23	1.29	0.009	0.0260
		M36429_s_at	GNB2	18	1.25	0.0020	0.0402
		U50648_s_at	ESTs	16	1.78	0.0013	0.0310

**Dor**	**MD**	X17093_at	HLA-F	50	1.37	0.0009	0.0293
		X06985_at	HMOX1	47	0.98	0.0013	0.0316
		U78556_at	MTMR11	42	1.05	0.0022	0.0378
		D14874_at	ADM	28	1.05	0.0016	0.0342
		D13900_at	ECHS1	27	1.05	0.0022	0.0376
	
	**MGlio**	HG919-HT919_at	ESTs	33	1.50	0.0003	0.0211
		U71598_at	ZNF274	30	1.61	0.0011	0.0316
		L40027_at	GSK3A	22	1.30	0.0100	0.0802
		L41939_at	EPHB2	15	1.10	0.0018	0.0353
		HG384-HT384_at	ESTs	14	1.23	0.0240	0.1233
	
	**Rhab**	U52828_s_at	CTNND2	42	2.07	3.25E-05	0.0193
		Y07829_xpt4_at	ESTs	33	1.79	2.19E-05	0.0173
		M37457_at	ESTs	28	1.75	4.32E-05	0.0171
		X99688_at	ESTs	21	1.89	3.56E-05	0.0181
		M14676_at	FYN	20	1.77	0.0001	0.0183
	
	**Ncer**	X04828_at	GNAI2	73	2.72	0	0
		HG2743-HT2846_s_at	ESTs	71	2.86	0	0
		HG2167-HT2237_at	ESTs	23	2.62	7.01E-06	0.0083
		HG3546-HT3744_s_at	ESTs	22	2.98	0.0001	0.0189
		X86018_at	LRRC41	21	3.65	3.61E-05	0.0172
	
	**PNET**	X13916_at	LRP1	39	1.44	0.0007	0.0268
		X60483_at	HIST1H4J	29	1.41	0.0015	0.0332
		U41816_at	PFDN4	27	1.25	0.0026	0.0411
		U25265_at	MAP2K5	26	1.54	0.0013	0.0313
		M12625_at	LCAT	23	1.13	0.0012	0.0314

Using the training data, for each class the top five most frequently occurring genes are selected.

**Table 4:** Details of the selected genes by the frequency-based method for the Lung Cancer data set

	Class	Probe ID	Gene Symbol	Frequency	Ave. GDI	*p*-value	*q*-value
**Dom**	**Adeno**	38261_at	ABCC3	100	0.84	2.68E-05	0.0005
		35276_at	CLDN4	77	0.66	0.0004	0.0038
		39339_at	TMEM63A	63	0.64	0.0005	0.0044
		1930_at	ABCC3	43	0.68	0.0006	0.0051
		1802_s_at	ERBB2	23	0.68	0.0053	0.0280
	
	**Normal**	36119_at	CAV1	99	2.13	0	0
		1815_g_at	ESTs	36	2.14	0	0
		40994_at	GRK5	35	2.00	0	0
		36569_at	CLEC3B	34	1.83	0	0
		1814_at	ESTs	33	1.75	0	0
	
	**SCLC**	893_at	UBE2S	52	2.64	0	0
		39990_at	ISL1	47	2.38	0	0
		32272_at	TUBA1B	47	2.05	0	0
		894_g_at	UBE2S	43	2.18	0	0
		39605_at	FOXG1	38	2.78	0	0
	
	**SQ**	613_at	KRT5	100	1.48	0	0
		31791_at	TP63	96	1.26	0	0
		1898_at	TRIM29	74	1.18	3.19E-07	1.61E-05
		39016_r_at	KRT6A	56	1.18	3.19E-07	1.51E-05
		41266_at	ITGA6	38	1.21	9.57E-07	4.21E-05
	
	**COID**	40825_at	MAPRE3	67	2.47	0	0
		32254_at	VAMP2	52	2.31	0	0
		40165_at	TSPYL2	49	2.36	0	0
		41107_at	SNPH	39	2.80	0	0
		198_g_at	NME3	26	2.31	0	0

**Dor**	**Adeno**	36209_at	BRD2	97	0.68	0.0008	0.0176
		39799_at	FABP5	74	0.66	0.0022	0.0305
		40580_r_at	PTMS	67	0.58	0.0037	0.0399
		1315_at	OAZ1	57	0.61	0.0038	0.0408
		39561_at	CBX6	39	0.59	0.0050	0.0474
	
	**Normal**	36133_at	DSP	55	1.73	0	0
		31850_at	GCLC	41	1.58	0	0
		1248_at	POLR2H	23	1.48	0	0
		35194_at	GPX2	23	1.46	0	0
		39353_at	HSPE1	20	2.29	6.38E-07	0.0003
	
	**SCLC**	33908_at	CAPN1	54	1.30	6.38E-07	0.0003
		1109_s_at	PDGFA	36	1.71	1.50E-05	0.0022
		36952_at	HADHA	27	1.28	1.26E-05	0.0019
		338_at	ATF6	25	1.32	5.70E-05	0.0046
		36890_at	PPL	20	1.32	4.55E-05	0.0042
	
	**SQ**	38113_at	SYNE1	34	1.51	4.90E-05	0.0043
		33118_at	SEMA3B	31	1.36	3.99E-06	0.0009
		40665_at	FMO3	26	1.48	0.0003	0.0115
		37908_at	GNG11	19	1.52	2.11E-05	0.0024
		33267_at	ATP8A1	19	1.22	8.14E-06	0.0015
	
	**COID**	33322_i_at	SFN	79	2.67	0	0
		39728_at	ESTs	39	1.75	0	0
		36879_at	ECGF1	35	2.24	0	0
		925_at	ESTs	25	1.64	0	0
		33143_s_at	SLC16A3	24	2.07	4.79E-07	0.0003

Using the training data, for each class the top five most frequently occurring genes are selected.

The WAS gene belongs to the set of Human Cancer Genes [18]. It has a very strong BL (Burkitt lymphomas) class specific signature, and it is also found important by others in the context of the SRBCT data set [19]. The relationship of WAS to Burkitt Lymphomas is also reported in [20]. The deficiency of WAS gene causes the Wiskott-Aldrich syndrome, which is an X-linked hereditary disease associating primary immunodeficiency, thrombocytopenia, an increased risk of autoimmune diseases and malignancies, particularly non-Hodgkin's lymphoma (NHL) [21-24]. In patients with Wiskott-Aldrich syndrome, a higher rate of malignancy has been observed, particularly in Epstein-Barr Virus-related brain tumor, leukemia and lymphoma . Amongst the different kinds of tumors, the most frequently associated one with Wiskott-Aldrich syndrome is the NHL tumor (it is about 76%). The other kinds of tumors associated with WAS include, Hodgkin's disease, glioma, and testicular carcinoma [21,24]. Although NHL is the most common type of malignancy found in WAS and BL represents 40% to 50% of all NHL cases in childhood, BL has hardly been reported in WAS. But a case of BL with WAS is reported in [20]. In [24], authors reported Malignant B Cell Non-Hodgkin's Lymphoma of the Larynx with Wiskott-Aldrich syndrome. All these clearly establishes the important role of WAS not only in BL, but in other types of malignancies too.

**Figure 1:**
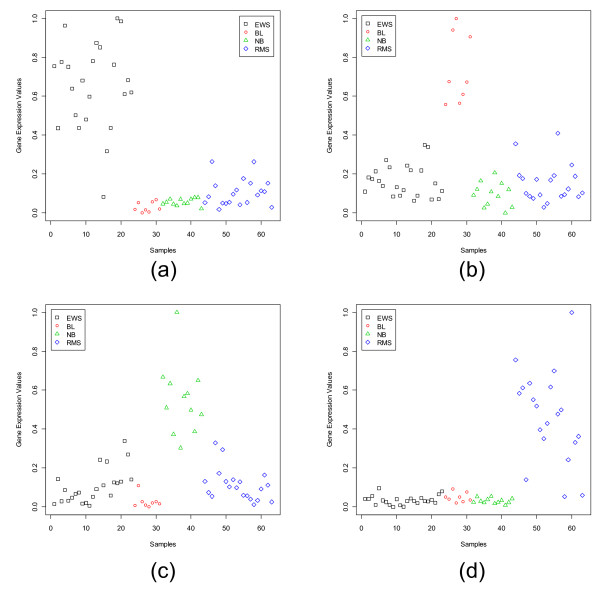
Scatterplots of the most dominant gene in each subgroup of the SRBCT data set: (a) FCGRT (Image: 770394) for EWS, (b) WAS (Image: 236282) for BL, (c) AF1Q (Image: 812105) for NB, (d) FGFR4 (Image: 784224) for RMS.

The ALL1-fused gene from chromosome 1q (AF1Q) is one of the dominant genes found by our method for the neuroblastoma (NB) group. Many authors have reported this gene to play important roles in cancer [25,26]. As revealed by Fig. 1(c), AF1Q is moderate to highly express for the neuroblastoma cases, while it exhibits low expression values for the other three groups of the SRBCT.

As discussed in [10,27], FGFR4 carries out the signal transduction to the intracellular environment in cellular proliferation, differentiation and migration. Overexpression of FGFR4 is found in various cancers, such as of pituitary, prostate, thyroid [28-30], but in normal tissues, FGFR4 expression is hardly noticeable. In our study with the SRBCT, we noticed a very strong RMS (rhabdomyosarcomas) specific signature, very high expression levels of FGFR4 for the RMS group, but for the other groups it is practically unexpressed. However, in lung adenoarcinoma, FGFR4 is found to be downregulated [31].

The second and third most dominant genes for the EWS class are Follicular lymphoma variant translocation 1 (FVT1) and CAV1 (caveolin 1, caveolae protein, 22 kD). According to [32] FVT1 is found to be weakly expressed in normal hematopoietic tissues, but is shown to exhibit a very high rate of transcription in some T-cell malignancies and in phytohemagglutinin-stimulated lymphocytes. Becuase of the proximity of FVT1 to BCL2, authors in [32] also have indicated that both genes may involve in the tumoral process. For the present data set, it exhibits a very strong EWS specific signature. Its expression is practically absent for RMS, NB and NHL groups, but it is highly expressed for the EWS group. The gene CAV1 is also a biologically informative gene. In our study we found CAV1 to be upregulated for the EWS group. According to [33], CAV1 is down-regulated in oncogene-transformed and tumor-derived cells and it is an essential structural constituent of caveolae that plays important roles in mitogenic signaling and oncogenesis. Many studies have reported CAV1 as a candidate tumor suppressor gene [34-36]. It has been established that CAV1 has tumor suppressor activity in human cancers, including breast cancer [33,37], ovarian cancer [38], and lung cancer [39]. But in [40], they showed that CAV1 is over-expressed in human gastric cancer cell line GTL-16. Also, for diffuse large B-cell lymphoma [41] and prostate cancer [42], CAV1 is identified to serve as a diagnostic and prognostic marker. For the Lung Cancer data set in this study we have found CAV1 as a good dominant gene for the normal tissue group and this is in conformity with the fact that CAV1 also plays the role of a tumor suppressor. This is also consistent with down-regulation of CAV1 in human lung carcinoma [39]. Thus, CAV1 plays an important role in cancer biology.

According to Table 2, we shall now discuss the importance of some dominant and dormant genes for the Leukemia data set. The MME (membrane metallo-endopeptidase), also known as CD10, is the most important dominant gene for the ALL group as found by *GDI*_*Dom*_. MME is found to play different roles in different types of cancers. In [43], authors suggested that the functions of MME vary with tissue types and disease states. For example, in hepatocellular and thyroid carcinoma MME exhibits higher expression levels [44,45], while in poorly differentiated tumors in the colon and stomach MME shows low expression levels [46]. According to [47], MME is downregulated in the ALL samples with MLL (Mixed-Lineage Leukemia) rearrangements compared to ALL without MLL rearrangements. In our study we have found MME to be highly expressed in ALL while for the MLL and AML groups it is moderately expressed.

The top two dominant genes for the MLL group found by GDI are MBNL1 and MEIS1. In our study, we have found that expression levels of both MBNL1 and MEIS1 are higher for the MLL group than the other two groups. In [48], authors have found upregulation of these two genes in the ALL and AML groups with MLL chimeric fusion genes. It is interesting to know that by just using three genes MME and MBNL1 and MEIS1, one can do a good job of discrimination between the three types of leukemia (results not shown); of course, three dominant genes, one from each class can do an excellent job of classification too.

The most dormant gene for the ALL class as detected by *GDI*_*Dor *_is LGALS1. In [49] it is claimed that a higher expression of LGALS1 is a negative prognostic predictor of recurrence in laryngeal squamous cell carcinomas. The next important dormant gene for the same class is ANXA1. This gene has been extensively studied and is found to play interesting roles in human cancers. Following [50,51] we summarize various cases where ANXA1 is up-regulated and down-regulated. Higher expression level of ANXA1 is observed in hepatocellular carcinoma [52], mammary adenocarcinoma [53], glioblastoma [54], and pancreatic cancer [55]. On the other hand, many investigations have reported down-regulation of ANXA1 in different types of cancers such as in the head and neck [56,57], esophageal [56], prostate [56], breast [50], and larynx [51]. In our study with the leukemia data set, ANXA1 is identified as a good dormant gene for the ALL group. Note that, an absence of ANAX1 expression is observed in B-cell non-Hodgkin's lymphomas too [58].

In our investigation with the CNS data set, as shown in Table 3, the transcriptional repressor, insulinoma-associated 1 (INSM1) is found to be one of the dominant genes for the MD (medulloblastomas) group. Different investigations have found this gene to play roles in tumors of neuroendocrine origin. In [59], they reported INSM1 as one of the important genes in discriminating pancreatic adenocarcinomas and islet cell tumors from normal pancreatic tissues. The gene INSM1 is also found to be over-expressed in small-cell lung cancer (SCLC), SCLC cell lines as well as in medullary thyroid carcinoma, insulinoma, and pituitary tumors [16,60,61].

As shown in Table 4, the most important dominant gene for the Adenocarcinoma group of the lung cancer data is ABCC3. The protein encoded by this gene belongs to the superfamily of ATP-binding cassette (ABC) transporters and is known to be involved in multi-drug resistance. The roles played by ABCC3 in different cancers are also reported in the literature [62-64]. For example, O'Brien et al. [62] have claimed that amplification and concomitant overexpression of the gene ABCC3 is responsible to confer resistance to paclitaxel and monomethyl-auristatin-E. Authors also demonstrated that this amplification is present in primary breast tumors. Benderra et al. [64] have suggested that ABCC3 may be involved in chemoresistance in AML. The GDI based method has identified Keratin 5 (KRT5) as the most dominant gene for the squamous cell lung carcinoma (SQ) group. An inspection of Fig. 7 reveals that for most of the SQ samples KRT5 is highly expressed while its expression level for the other four groups in the Lung Cancer data set is practically absent. This strong SQ specific signature of KRT5 is also reported in [65,66].

### Visual assessment of the dominant/dormant marker genes

In the next section we shall demonstrate the utility of the identified genes through performance comparison with different classifiers. But classifier performance is an indirect indicator. It does not reveal how dominant (dormant) a gene is with respect to a class. So we try to make visual assessments of the quality of the dominant (dormant) genes. For this we adopt two approaches. First, we use scatterplots to view the distribution of the expression values of a dominant (dormant) gene in all samples (not including samples of the independent data set). This helps us to assess the discriminating power of (each) *individual *gene. Second, we try to visualize the overall discriminating power of *a set of *dominant (dormant) genes selected based on GDIs. This is done by looking at a two-dimensional plot generated using Sammon's Non-linear Projection [67] that preserves the inter-point distances in the high dimensional space. Note that, Sammon's method does not use class information. The plots are labeled using the class information just for better visualization. For the Leukemia and SRBCT data sets, in the Sammon's plot we include both the training and independent data sets (for the training data different classes are represented by different shapes with different colors; for the independent test data, the same shapes are used but filled in with colors).

**Figure 2:**
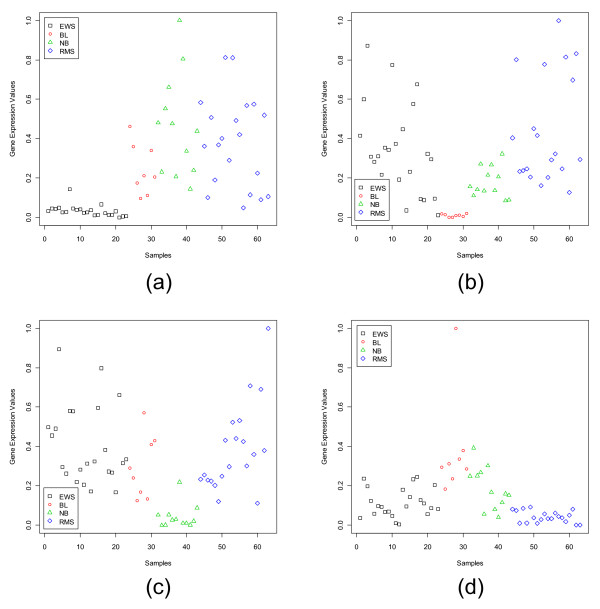
Scatterplots of the most dormant gene in each subgroup of the SRBCT data set: (a) CDK6 (Image: 295985) for EWS, (b) ANTXR1 (Image: 204545) for BL, (c) CSDA (Image: 810057) for NB, (d) PGAM1 (Image: 897177) for RMS.

**Figure 3:**
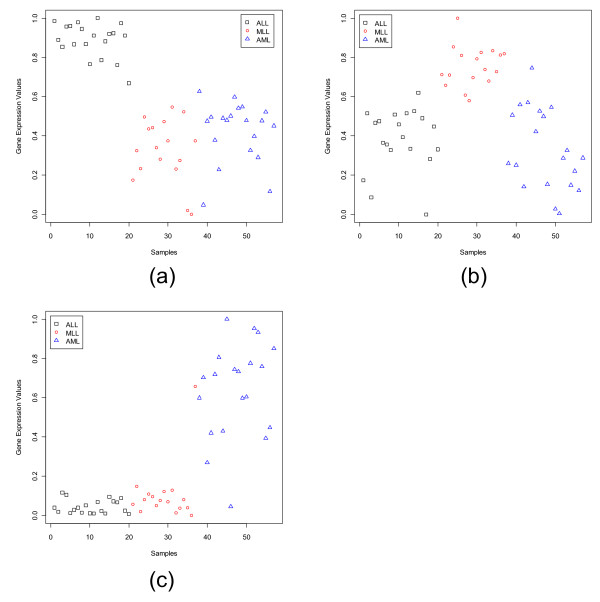
S**catterplots of the most dominant gene in each subgroup of the Leukemia data set: (a) MME (1389_at) for ALL, (b) MBNL1 (34306_at) for MLL, (c) CHRFAM7A (39566_at) for AML.**

**Figure 4:**
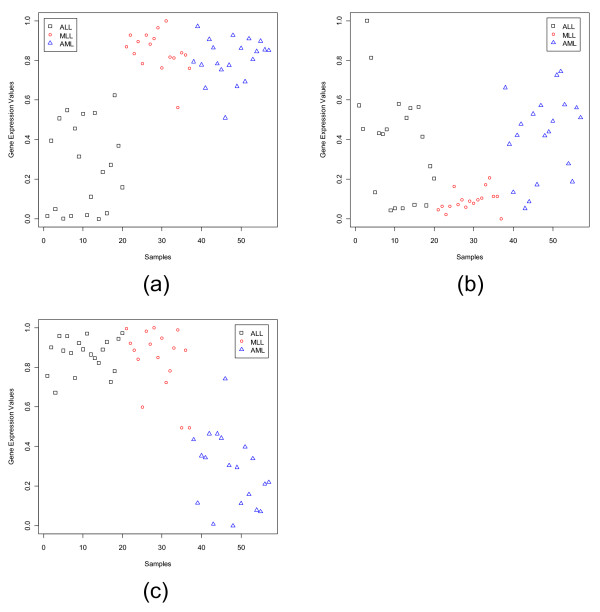
Scatterplots of the most dormant gene in each subgroup of the Leukemia data set: (a) LGALS1 (33412_at) for ALL, (b) YES1 (1674_at) for MLL, (c) MEF2A (41747_s_at) for AML.

**Figure 5:**
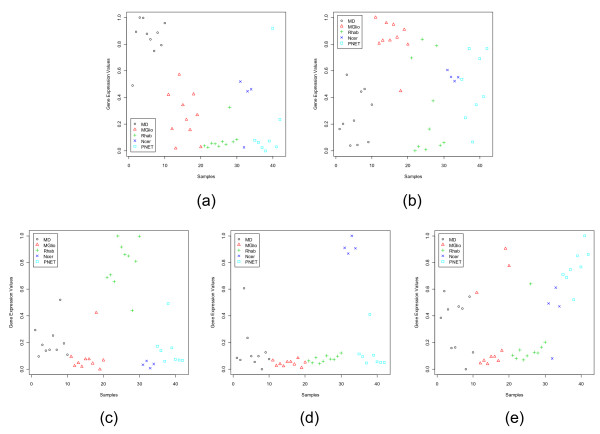
Scatterplots of the most dominant gene in each subgroup of the CNS data set: (a) INSM1 (M93119_at) for MD, (b) PTPRZ1 (M93426_at) for MGlio, (c) SLC35A2 (D84454_at) for Rhab, (d) GRM4 (U92457_s_at) for Ncer, (e) IGHD (K02882 cds1_s_at) for PNET.

**Figure 6:**
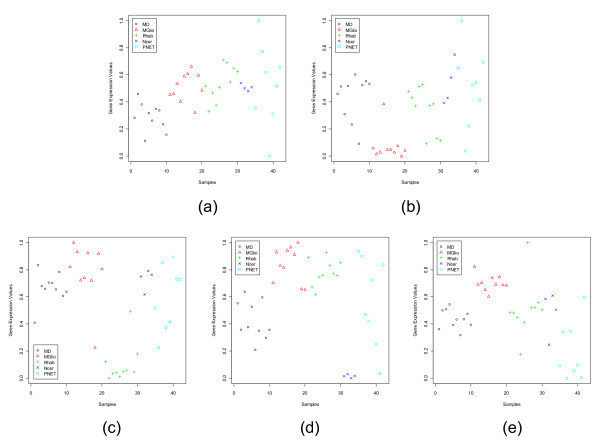
Scatterplots of the most dormant gene in each subgroup of the CNS data set: (a) HLA-F (X17093_at) for MD, (b) ESTs (HG919-HT919_at) for MGlio, (c) CTNND2 (U52828_s_at) for Rhab, (d) GNAI2 (X04828_at) for Ncer, (e) LRP1 (X13916_at) for PNET.

**Figure 7:**
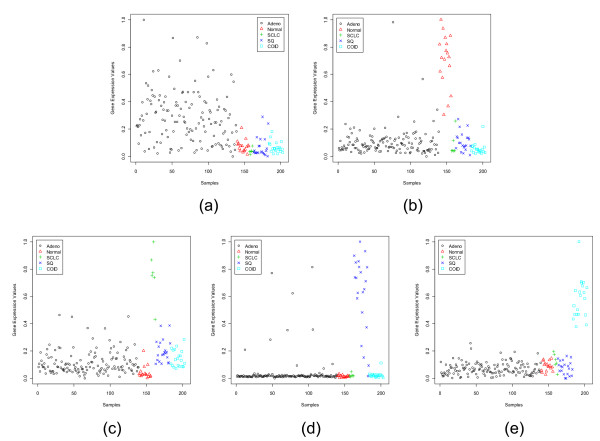
Scatterplots of the most dominant gene in each subgroup of the Lung Cancer data set: (a) ABCC3 (38261_at) for Adeno, (b) CAV1 (36119_at) for Normal, (c) UBE2S (893_at) for SCLC, (d) KRT5 (613_at) for SQ, (e) MAPRE3 (40825_at) for COID.

**Figure 8:**
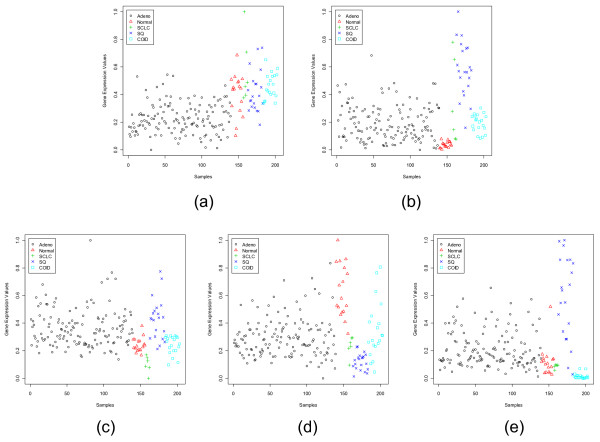
Scatterplots of the most dormant gene in each subgroup of the Lung Cancer data set: (a) BRD2 (36209_at) for Adeno, (b) DSP (36133_at) for Normal, (c) CAPN1 (33908_at) for SCLC, (d) SYNE1 (38113_at) for SQ, (e) SFN (33322_i_at) for COID.

In Figs. 1, 2, 3, 4, 5, 6, 7, 8, the y-axis expresses the observed gene expression values (normalized in [0,1]), the x-axis indicates the samples in a data set. The samples in different groups (classes) are represented by different symbols and colors. The four panels in Fig. 1 display the four most dominant (one for each class) genes for the SRBCT data set. As expected, the dominant gene for a class appears with high expression values in the samples from that class, but with low expression values in the samples of the other classes/subgroups. As an example, for the SRBCT data set the most dominant gene, FCGRT (Image: 770394), for the Ewing Sarcoma is highly expressed for the EWS group while, it is practically unexpressed for the other three SRBCT classes (Fig. 1(a)). Similarly, Fig. 1(b) shows that for the Burkitt Lymphomas (BL) the most dominant gene, WAS (Image: 236282), is over-expressed for the BL samples but under-expressed for the other classes.

Figure 2 depicts that for the SRBCT the dormant genes for all four classes are not very good and that explains the poor performance of the classifiers discussed later. In Fig. 2 we find that the most dormant gene, CDK6 (Image: 295985), for the EWS is completely unexpressed for the EWS samples while it is moderately expressed for the remaining three classes. Of the remaining three classes, the average expression level for the BL group is the closest to that of EWS group. Although, from pattern recognition point of view, this gene can distinguish EWS from the other three classes, since the difference between the average expression levels for EWS and BL groups is not high, this gene may not be considered a very good dormant gene. In some cases, the identified dormant genes may not even be good from pattern recognition point of view also. As an example, consider Fig. 2(d) depicting the expression values of the most dormant gene for the RMS group. Clearly, the distribution of expression levels reveals that this gene cannot distinguish the RMS group from the EWS and NB groups. This is an indicator that for the RMS group we do not have any good dormant gene. This can be checked from the average values of *GDI*_*Dor *_in Table 1. For the EWS and BL groups the average *GDI*_*Dor *_values for the most dormant genes are 1.37 and 2.04 respectively, while for the RMS group it is only 0.75.

The scatterplots of three most dominant genes for the Leukemia data set, one for each class, are displayed in Fig. 3. Fig. 3(a) depicts that the gene MME has a very strong ALL specific signature and Fig. 3(c) representing CHRFAM7A has a strong signature for the AML group; while the gene MBNL1 (Fig. 3(b)) although has an MLL specific signature, it is not as strong as that of the other two genes. Fig. 4 depicts the scatterplots of the most dormant genes for Leukemia data set. Here we find that for majority of the samples in the ALL group, the most dormant gene, LGALS1, takes low expression values compared to the samples from the other two groups. In this case the separation between the average expression values between the ALL and AML groups is quite high making it a good dormant gene. This is also revealed by the GDI values of 1.66. Similarly, for the AML class, the most dormant gene, MEF2A, is downregulated for the AML group, while it is upregulated for the remaining groups (the average GDI value is 1.89). Thus, this gene can also be considered a good dormant gene.

Figures 5 and 6 display the scatterplots of the dominant and dormant genes, respectively, for the CNS data set while Figs. 7 and 8 depict the same for the Lung Cancer data set. The Lung Cancer data set have five subgroups. Except for the adenocarcinoma group, each of the remaining subgroups has a dominant gene with very strong group specific signature. The adenocarcinoma group has the largest number of samples. Although, on average the dominant gene for this group has a higher expression level, there are several samples with low expression values too.

**Figure 9:**
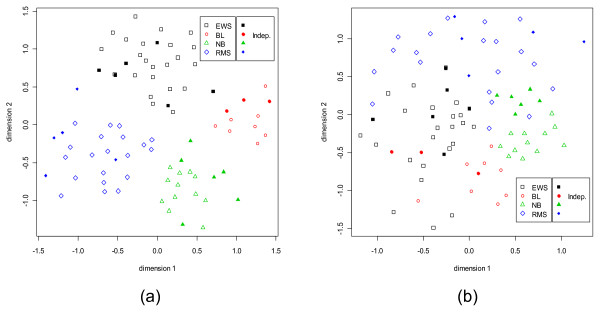
**Sammon's plots for the SRBCT data set using the training and independent data together.** For the training data different classes are represented by different shapes with different colors. For the independent test data, the same shapes are used but filled in with colors; e.g., the training data from the EWS class is represented by black empty square and the test data from the same class, EWS, is represented by filled in black square. (a) With 5 dominant genes from every class. (b) With 5 dormant genes from every class.

**Figure 10:**
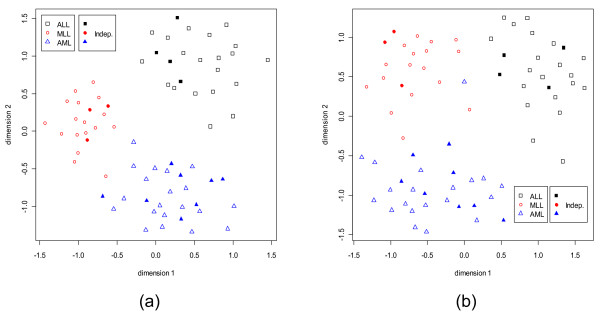
S**ammon's plots for the Leukemia data set using the training and independent data together.** For the training data different classes are represented by different shapes with different colors. For the independent test data, the same shapes are used but filled in with colors; e.g., the training data from the ALL class is represented by black empty square and the test data from the same class, ALL, is represented by filled in black square. (a) With 5 dominant genes from every class. (b) With 5 dormant genes from every class.

**Figure 11:**
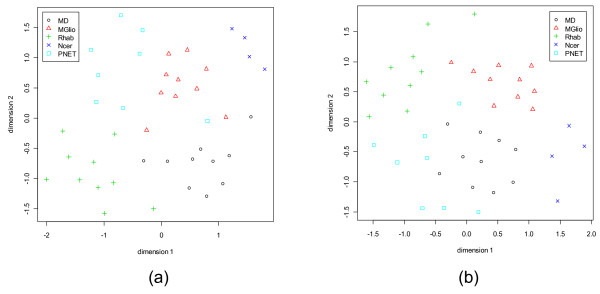
**Sammon's plots for the CNS data set.** Different classes are represented by different shapes with different colors. (a) With 5 dominant genes from every class. (b) With 5 dormant genes from every class.

**Figure 12:**
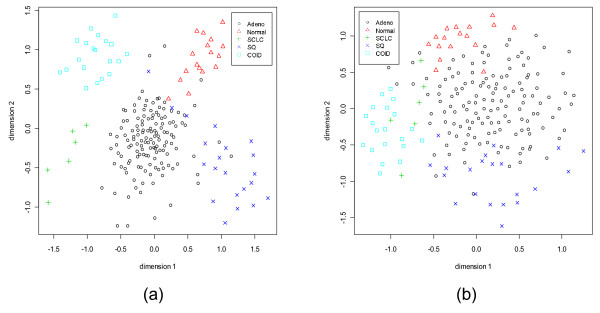
**Sammon's plots for the Lung Cancer data set.** Different classes are represented by different shapes with different colors. (a) With 5 dominant genes from every class. (b) With 5 dormant genes from every class.

Now we shall analyze sets of genes selected by our method using Sammon's Projection (Figs. 9, 10, 11, 12). We use the function "sammon" in MASS library in R  in conjunction with random initial configuration. For each class we select all top five selected dominant genes. For example, in case of SRBCT we have used 20 dominant genes, five from each of the four classes. For the scatterplots we have used the normalized expression values for an easy visual assessment, but here since we want to preserve inter-point distances, we use the data obtained after preprocessing. For the SRBCT data set, the Sammon's plot is shown in Fig. 9(a). In Fig. 9(a), for the training data different classes are represented by different shapes with different colors. For the independent test data, we use the same shapes but filled in with colors. For example, if the training data from a class is represented by red empty square, then the test data from the same class will be represented by filled in red square. Figure 9(a) reveals that samples from different classes form nice clusters both for the training and *independent *data sets, although the gene selection is done exclusively based on the training set. Figure 9(b) depicts the Sammon's plot using the dormant genes. Comparing the Sammon's plot with the dominant genes, we find that although the dormant genes approximately reveal the class structures, these are not as clear as in the case of the dominant genes. In fact, there are some mixing up of the groups. This explains the poor test performance obtained with the dormant genes (details in the next section).

For the Leukemia data set, Figs. 10(a) and 10(b) display the Sammon's plots using dominant and dormant genes considering the training and *independent *data sets together. Unlike, SRBCT here for both dominant and dormant genes the three classes are almost well separated. This is in conformity with comparable and good performance of all six classifiers using the dominant and dormant genes (discussed in the next section). These results imply that the dominant or dormant genes selected from each subgroup of the microarray data set contribute good discrimination power between classes.

For the CNS and the Lung Cancer data sets there is no independent test data set. Figs. 11 and 12 show the Sammon's plots for these two data sets. In the case of CNS, with dominant genes, the Sammon's plot exhibits very nice class structure for all classes (only one point of PNET, primitive neuro-ectodermal tumors, class is mixed up). But for the dormant genes, all but PNET class form nice clusters in the Sammon's plot. For the Lung Cancer data although with the dominant genes the class structures emerge in the Sammon's plot, with the dormant genes the COID (pulmonary carcinoids) group stands out separately but other classes are overlapped. This should not be used to infer that the performance of classifiers using the dormant genes would be poor – this is not indeed the case. In the next section, we will demonstrate that even with the dormant genes all six classifiers perform quite well. This might mean that if we would use a higher dimensional Sammon's plot we might obtain a better separability between classes.

### Comparison of classifier performance

**Figure 13:**
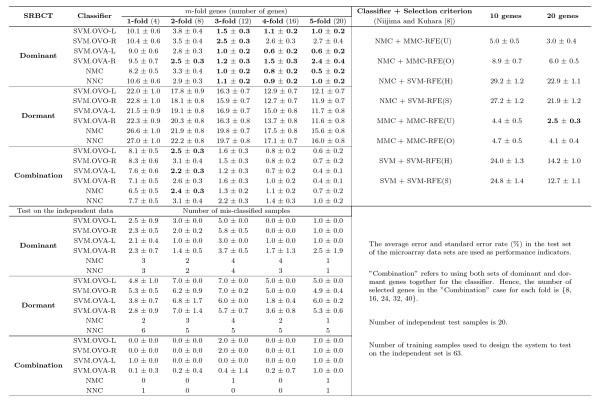
**Evaluation of performance of six classifiers using different number of dominant genes, dormant genes and their combination for the SRBCT data set along with its comparison with the results reported in**[8]. The performance of the proposed methods on the independent test data is also included. Here *m*-fold corresponds to the case when *m *top most dominant (dormant) genes are used for each class. For example, the column labeled 3-fold represents the results using 12 genes (3 dominant (dormant) genes from each of the 4 classes) for the SRBCT data set.

**Figure 14:**
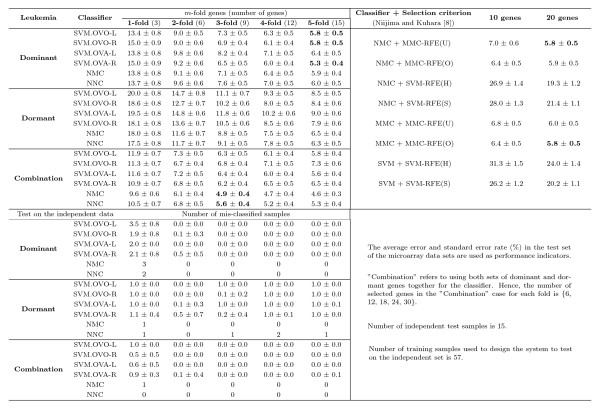
**Evaluation of performance of six classifiers using different number of dominant genes, dormant genes and their combination for the Leukemia data set along with its comparison with the results reported in**[8]. The performance of the proposed methods on the independent test data is also included. Here *m*-fold corresponds to the case when *m *top most dominant (dormant) genes are used for each class. For example, the column labeled 3-fold represents the results using 9 genes (3 dominant (dormant) genes from each of the 3 classes) for the Leukemia data set.

**Figure 15:**
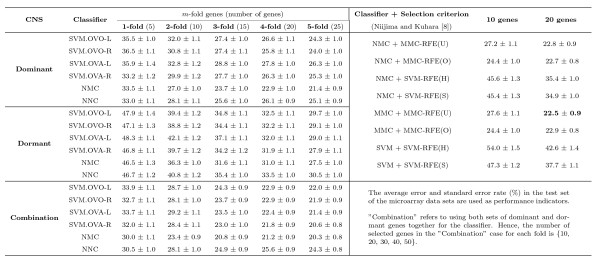
**Evaluation of performance of six classifiers using different number of dominant genes, dormant genes and their combination for the CNS data set along with its comparison with the results reported in**[8]. Here *m*-fold corresponds to the case when *m *top most dominant (dormant) genes are used for each class. For example, the column labeled 3-fold represents the results using 15 genes (3 dominant (dormant) genes from each of the 5 classes) for the CNS data set.

**Figure 16:**
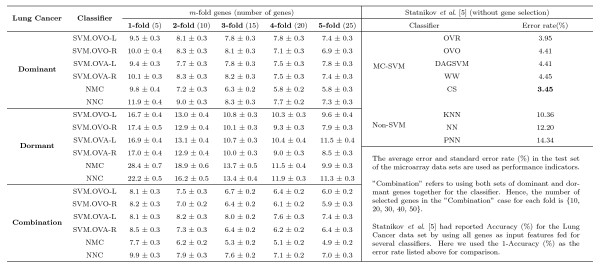
**Evaluation of performance of six classifiers using different number of dominant genes, dormant genes and their combination for the Lung Cancer data set along with its comparison with the results reported in**[5]. In [5] all genes were used. Here *m*-fold corresponds to the case when *m *top most dominant (dormant) genes are used for each class. For example, the column labeled 3-fold represents the results using 15 genes (3 dominant (dormant) genes from each of the 5 classes) for the Lung Cancer data set.

We conduct our experiments to examine the results using six distinct classifiers (three of them are used in [8]) with different number of dominant or dormant genes selected by our method for the SRBCT, Leukemia, CNS, and Lung Cancer data sets. In our frequency based method we select the top five dominant (dormant) genes for each class in 100 simulations, and then determine the frequency with which these genes appear as the dominant (dormant) candidates for that class. A more detailed discussion is set forth in Materials and Methods. Figs. 13, 14, 15, 16 summarize the performance of the proposed method for the four data sets SRBCT, Leukemia, CNS, and Lung Cancer respectively. In these figures we summarize the results as follows: For a *k*-class problem, for each class we use *m *number of genes, with *m *= 1, 2, ⋯, 5. When *m *= 1, we call it 1-fold case, *m *= 2 is called the 2-fold case and so on.

On the right side of Figs. 13, 14, 15, for an easy reference, we also include the relevant summary of the prediction results in [8] using different gene selection methods. Here we display the prediction result in bold if it is better than the best classification error reported in [8] and uses less (or equal) number of genes than that in [8]. For the SRBCT data set, with only three dominant genes from each class, the performance of all six classifiers are better than the best performance reported by Niijima et al. [8] using 20 genes by their eight classifiers (as shown in Fig.13). This may be taken as an indicator of strong dominancy of the selected genes. On the other hand, the performance of the dormant genes are not very good signifying absence of good dormant genes which is also confirmed by Fig. 9(b). Although, the performance of the dormant genes are not very good, the performance of our four SVM classifiers with 20 (*m *= 5) dormant genes is better than that by the four classifiers (SVM + SVM-RFE (H), SVM + SVM-RFE (S), NMC + SVM-RFE (H), NMC + SVM-RFE (S) [8] using 10 and 20 genes, respectively.

For the Leukemia data set, Fig. 14 reveals, with 15 genes all of our six classifiers yield very comparable (or marginally better) than the best result reported in [8] using 20 selected genes. Note that, in [8] using 20 genes the best classification error achieved on the test data is 5.8%; while in our case even with just 12 dominant genes all six classifiers can produce very comparable test accuracies with that of the best results in [8] using 20 genes. The performance of our four SVM classifiers using just 3 genes (one from each class) is better than that of both SVM classifiers in [8] using 20 genes. This clearly indicates the quality of the dominant marker genes identified by *GDI*_*Dom*_. Unlike the SRBCT, for this data set, the dormant genes also have good discriminating power. In fact, with 15 dormant genes the classification error rates for our two non-SVM classifiers are comparable to the best classier performance in [8] using 20 genes; while the performance of our four SVM classifiers is significantly better than that of the remaining four classifiers in [8]. In these figures "Combination" refers to using both sets of dominant and dormant genes together to design the classifier. In Fig. 14, with 18 genes (3-fold, 9 dominant and 9 dormant genes), the lowest error rate of 4.9% is achieved. Here we observe that combining dominant and dormant genes does not always improve the performance of the classifiers. However, later we shall see that use of dominant and dormant genes together improves the performance on the independent test data.

In [8] authors proposed two new gene selection methods based on MMC and used two SVM based gene selection methods from the literature. Considering three classifiers NMC, MMC, and SVM, they have reported results using eight combinations of classifier and gene selection method as shown in the right side of Fig. 15. For each of these eight combinations they have considered 10 genes and 20 genes for performance evaluation. Considering the combinations using the SVM based gene selection and the NMC and SVM classifiers, for the CNS data we find that the test error varies between 45.4% and 54.0% using 10 genes, while the same lies between 34.9% and 42.6% using 20 genes. On the other hand, using the MMC based feature selection methods, the error rates for the NMC and MMC classifiers using 10 genes vary between 24.4% and 27.6%, while error rates using 20 genes lie in 22.5%–22.9%. We observe in Fig. 15 that using *just 5 *dominant genes, one from each class identified by our method, the error rates of the six classifiers varied between 33% and 36%, while using 20 dominant genes the test error rates over the six classifiers varied between 22.9% and 27.8%.

Since there is an independent data set for each of the SRBCT and Leukemia, we have used the selected dominant/dormant genes in Table 1 and Table 2 to examine the prediction performance on those independent data sets. For these two data sets, *all *samples in the training data are used to train different classifiers using the selected genes with *m *= 1 to 5 folds. Then the trained classifiers are used to evaluate their performance on the independent test data set. Here we have normalized the expression value of each gene to [0,1] across samples considering both training and independent data sets. Note that, for the SVM classifier we need to choose some hyper-parameters. As done for other experiments, the training data set is randomly divided into training and validation sets of equal size. Then the validation set is used to choose the hyper-parameters. The classifier thus designed is tested on the independent data set. Like other experiments, here too the training-validation partition is repeated 100 times and the average number of misclassification and its standard deviation on the independent test data are reported in Figs. 13 and 14. From Fig. 13 we find that even just with 4 dominant genes the performance of all classifiers on the independent test set is quite good. The effect of the use of combined gene is very prominent for the SRBCT data set. For all folds 1 to 5, the performance of all classifiers on the independent test set is excellent. For the Leukemia data set also with just 3 dominant genes, the six classifiers make 2–3 mistakes and with just six genes all six classifiers result in around zero misclassification on the independent test data (Fig. 14). The classification performance of the dormant genes on the independent data is very good too. In this case, the performance of all six classifiers with 3 dormant genes is better than the performance of the classifiers with 3 dominant genes. For this data set, the performance of all six classifiers using dominant and dormant genes together on the test data is excellent too.

In Fig. 16, we examine the prediction performance for the Lung Cancer data set (not used in [8]) using the same six classifiers with different number of dominant or dormant genes selected by the proposed method. For this data set we compare our results with those in [5]. In [5] three non-SVM classifiers and five SVM classifiers are used. Figure 16 reveals that for three non-SVM classifiers (KNN, NN and PNN) using all 12600 genes, the prediction errors reported in [5] vary between 10.36% ~14.34%, while using *just 5 *dominant genes, with one gene per class, the performance of our six classifiers are quite good and are comparable or better than that of the three non-SVM classifiers. With just 20 dominant genes (four genes per class), the test error rates of our six classifiers vary between 5.8% and 7.8% while the best accuracy reported in [5] is 3.35% but the method in [5] use all 12600 genes. Here although our best result is about 2~3% lower than that of the best result in [5] (see, far right side of Fig. 16), the evaluation criteria and computational protocols are *not *the same. For example, we have used *only *5–25 (less that 0.20% of the 12600) genes, while in [5]*all *12600 genes are used; we have generated statistics about test accuracies using 100 sets (generated by resampling), while in [5] a 10 fold cross-validation is used; for the SVM classifier we have used the most simple linear kernel and the nonlinear Gaussian kernel for comparison, while in [5] authors have used nonlinear polynomial kernel and several other sophisticated classifiers such as back-propagation neural networks, and probabilistic neural networks.

In order to look at the statistical significance of the average GDI values of the dominant and dormant genes identified based on 100 data splitting experiments, here we further perform the permutation test 500 times (Details about the procedure can be found in the Materials and Methods section). These results are summarized in Tables 1, 2, 3, 4. From these tables we find that each of the selected dominant/dormant genes in every data set has a highly reliable *p*- and *q*-values. Especially, for those selected dominant/dormant genes, which appeared with very high frequencies, the *p*- and *q*-values are practically zero (0). Hence, from a statistical viewpoint, our method can recognize genes with trustworthy class-specific characteristics. Such genes can be used to design more reliable diagnostic systems.

In addition, we have checked the literature for other similar methods for identifying marker genes associated with one class in a multiclass environment. In this context, Pavlidis and Noble [11] use ANOVA and Correlation together. We call this scheme as ANOVA+Correlation scheme. In [12] SNR is used for preliminary screening of genes which is followed by the use of a SVM based technique. Both these schemes for multiclass analysis use a one-versus-all (OVA) approach. We have implemented the ANOVA+Correlation scheme and also used SNR with OVA strategy to select class specific genes. The later method is referred to as "OVA.SNR". As revealed by Tables 5 and 6, all three gene selection methods (GDI.Dominant, OVA.SNR and ANOVA+Correlation) produce comparable results.

**Table 5:** Comparison of performance for the SRBCT and Leukemia data sets using six classifiers with the same number of genes chosen by three gene selection methods

Data Sets	Gene Selection Methods	Classifiers	*m*-fold of genes				
			
			1-fold	2-fold	3-fold	4-fold	5-fold
SRBCT	GDI.Dominant	OVO.SVM-L	10.1 ± 0.6	3.8 ± 0.4	1.5 ± 0.3	1.1 ± 0.2	1.0 ± 0.2
		OVO.SVM-R	10.4 ± 0.6	3.5 ± 0.4	2.5 ± 0.3	2.6 ± 0.3	2.7 ± 0.4
		OVA.SVM-L	9.0 ± 0.6	2.8 ± 0.3	1.0 ± 0.2	0.6 ± 0.2	0.6 ± 0.2
		OVA.SVM-R	9.5 ± 0.7	2.5 ± 0.3	1.2 ± 0.3	1.5 ± 0.3	2.4 ± 0.4
		NMC	8.2 ± 0.5	3.3 ± 0.4	1.0 ± 0.2	0.8 ± 0.2	0.5 ± 0.2
		NNC	10.6 ± 0.6	2.9 ± 0.3	1.1 ± 0.2	0.9 ± 0.2	1.0 ± 0.2
	
	OVA.SNR [12]	OVO.SVM-L	11.1 ± 0.7	3.8 ± 0.4	1.3 ± 0.3	0.9 ± 0.3	0.8 ± 0.3
		OVO.SVM-R	11.8 ± 0.8	4.0 ± 0.5	3.2 ± 0.4	3.8 ± 0.5	3.5 ± 0.5
		OVA.SVM-L	9.5 ± 0.7	3.2 ± 0.4	1.1 ± 0.3	0.7 ± 0.2	0.6 ± 0.2
		OVA.SVM-R	10.7 ± 0.7	3.6 ± 0.5	1.9 ± 0.4	2.6 ± 0.4	3.0 ± 0.4
		NMC	9.2 ± 0.6	3.9 ± 0.4	1.1 ± 0.2	0.9 ± 0.2	0.5 ± 0.2
		NNC	10.4 ± 0.6	3.4 ± 0.4	1.2 ± 0.3	0.9 ± 0.2	0.8 ± 0.2
	
	ANOVA+Correlation [11]	OVO.SVM-L	10.7 ± 0.6	3.4 ± 0.4	1.4 ± 0.3	0.6 ± 0.2	0.8 ± 0.2
		OVO.SVM-R	10.8 ± 0.6	3.6 ± 0.4	2.1 ± 0.4	1.9 ± 0.4	1.8 ± 0.4
		OVA.SVM-L	9.4 ± 0.6	2.8 ± 0.4	1.2 ± 0.3	0.4 ± 0.2	0.5 ± 0.2
		OVA.SVM-R	8.8 ± 0.6	2.9 ± 0.4	1.6 ± 0.3	1.5 ± 0.3	1.4 ± 0.3
		NMC	8.1 ± 0.6	3.1 ± 0.4	0.9 ± 0.2	0.5 ± 0.2	0.6 ± 0.2
		NNC	10.0 ± 0.5	3.0 ± 0.4	1.0 ± 0.2	0.5 ± 0.2	0.7 ± 0.2

Leukemia	GDI.Dominant	OVO.SVM-L	13.4 ± 0.8	9.0 ± 0.5	7.3 ± 0.5	6.3 ± 0.5	5.8 ± 0.5
		OVO.SVM-R	15.0 ± 0.9	9.0 ± 0.6	6.9 ± 0.4	6.1 ± 0.4	5.8 ± 0.5
		OVA.SVM-L	13.8 ± 0.8	9.8 ± 0.6	8.2 ± 0.4	7.1 ± 0.5	6.4 ± 0.5
		OVA.SVM-R	15.0 ± 0.9	9.2 ± 0.6	6.5 ± 0.5	6.0 ± 0.4	5.3 ± 0.4
		NMC	13.8 ± 0.8	9.1 ± 0.6	7.1 ± 0.5	6.4 ± 0.5	5.9 ± 0.4
		NNC	13.7 ± 0.8	9.6 ± 0.6	7.6 ± 0.5	7.0 ± 0.5	6.0 ± 0.5
	
	OVA.SNR [12]	OVO.SVM-L	13.4 ± 0.8	9.1 ± 0.6	7.5 ± 0.5	7.0 ± 0.5	6.4 ± 0.4
		OVO.SVM-R	13.5 ± 0.8	8.8 ± 0.7	7.2 ± 0.5	6.6 ± 0.4	6.4 ± 0.5
		OVA.SVM-L	14.2 ± 0.8	10.9 ± 0.7	8.4 ± 0.5	7.2 ± 0.5	6.8 ± 0.4
		OVA.SVM-R	13.6 ± 0.8	9.5 ± 0.6	7.2 ± 0.5	6.4 ± 0.4	6.1 ± 0.5
		NMC	15.0 ± 0.7	8.8 ± 0.5	7.0 ± 0.5	6.7 ± 0.5	6.4 ± 0.5
		NNC	13.5 ± 0.7	8.7 ± 0.5	7.8 ± 0.5	7.7 ± 0.5	7.2 ± 0.5
	
	ANOVA+Correlation [11]	OVO.SVM-L	12.9 ± 0.8	10.2 ± 0.6	8.1 ± 0.5	7.9 ± 0.5	7.1 ± 0.5
		OVO.SVM-R	13.5 ± 0.8	10.0 ± 0.7	7.7 ± 0.5	7.1 ± 0.5	6.9 ± 0.5
		OVA.SVM-L	12.7 ± 0.8	11.6 ± 0.6	9.3 ± 0.5	8.5 ± 0.5	7.6 ± 0.5
		OVA.SVM-R	12.7 ± 0.8	9.8 ± 0.6	8.0 ± 0.6	6.8 ± 0.5	6.4 ± 0.5
		NMC	14.5 ± 0.8	9.4 ± 0.6	7.8 ± 0.6	7.1 ± 0.5	6.6 ± 0.5
		NNC	12.3 ± 0.7	9.8 ± 0.6	8.8 ± 0.6	7.4 ± 0.5	6.9 ± 0.5

Here *m*-fold corresponds to the case when *m *top most dominant genes are used for each class. For example, the column labeled 3-fold represents the results using 12 genes (3 dominant genes from each of the 4 classes) for the SRBCT data set. Similarly, for the Leukemia data set the same column represents results using 9 dominant genes as there are 3 classes.

**Table 6:** Comparison of performance for the CNS and Lung Cancer data sets using six classifiers with the same number of genes chosen by three gene selection methods

Data Sets	Gene Selection Methods	Classifiers	*m*-fold of genes				
			
			1-fold	2-fold	3-fold	4-fold	5-fold
CNS	GDI.Dominant	OVO.SVM-L	35.5 ± 1.0	32.0 ± 1.1	27.4 ± 1.0	26.6 ± 1.1	24.3 ± 1.0
		OVO.SVM-R	36.5 ± 1.1	30.8 ± 1.1	27.4 ± 1.1	25.8 ± 1.1	24.0 ± 1.0
		OVA.SVM-L	35.9 ± 1.4	32.8 ± 1.2	28.8 ± 1.0	27.8 ± 1.0	26.3 ± 1.0
		OVA.SVM-R	35.2 ± 1.2	29.9 ± 1.2	27.7 ± 1.0	26.3 ± 1.0	25.3 ± 1.0
		NMC	33.5 ± 1.1	27.0 ± 1.0	23.7 ± 1.0	22.9 ± 1.0	21.4 ± 0.9
		NNC	33.0 ± 1.1	28.1 ± 1.1	25.6 ± 1.0	26.1 ± 0.9	25.1 ± 0.9
	
	OVA.SNR [12]	OVO.SVM-L	37.1 ± 1.1	30.5 ± 1.0	27.0 ± 1.0	23.5 ± 1.0	21.6 ± 1.0
		OVO.SVM-R	35.9 ± 1.1	29.6 ± 1.0	27.1 ± 1.0	23.2 ± 1.0	21.6 ± 1.0
		OVA.SVM-L	36.8 ± 1.1	30.9 ± 1.1	26.8 ± 0.9	22.8 ± 0.9	20.8 ± 0.9
		OVA.SVM-R	35.2 ± 1.1	29.1 ± 0.9	26.1 ± 0.9	23.7 ± 0.9	21.6 ± 1.0
		NMC	32.8 ± 1.1	26.3 ± 1.0	23.5 ± 1.0	20.6 ± 0.8	18.5 ± 0.9
		NNC	34.9 ± 1.0	28.3 ± 1.0	26.0 ± 1.0	24.0 ± 0.9	21.5 ± 1.0
	
	ANOVA+Correlation [11]	OVO.SVM-L	38.5 ± 1.1	32.2 ± 1.0	27.0 ± 0.9	24.0 ± 0.8	21.6 ± 0.8
		OVO.SVM-R	37.1 ± 1.1	30.8 ± 1.0	27.4 ± 1.0	25.0 ± 0.9	21.4 ± 0.8
		OVA.SVM-L	38.5 ± 1.1	33.4 ± 1.0	27.4 ± 1.0	25.5 ± 0.8	23.2 ± 0.8
		OVA.SVM-R	35.8 ± 1.1	31.0 ± 1.1	26.0 ± 1.0	25.1 ± 0.8	24.1 ± 0.9
		NMC	33.7 ± 1.3	24.9 ± 0.9	20.8 ± 0.8	19.7 ± 0.8	19.2 ± 0.8
		NNC	36.0 ± 1.2	29.7 ± 1.0	24.4 ± 0.9	23.3 ± 0.8	21.4 ± 0.7

Lung Cancer	GDI.Dominant	OVO.SVM-L	9.5 ± 0.3	8.1 ± 0.3	7.8 ± 0.3	7.8 ± 0.3	7.4 ± 0.3
		OVO.SVM-R	10.0 ± 0.4	8.3 ± 0.3	8.1 ± 0.3	7.1 ± 0.3	6.9 ± 0.3
		OVA.SVM-L	9.4 ± 0.3	7.7 ± 0.3	7.8 ± 0.3	7.5 ± 0.3	7.8 ± 0.3
		OVA.SVM-R	10.1 ± 0.3	8.3 ± 0.3	8.2 ± 0.3	7.5 ± 0.3	7.4 ± 0.3
		NMC	9.8 ± 0.4	7.2 ± 0.3	6.3 ± 0.2	5.8 ± 0.2	5.8 ± 0.3
		NNC	11.9 ± 0.4	9.0 ± 0.3	8.3 ± 0.3	7.7 ± 0.2	7.3 ± 0.3
	
	OVA.SNR [12]	OVO.SVM-L	9.8 ± 0.3	8.0 ± 0.3	8.1 ± 0.3	8.0 ± 0.3	7.4 ± 0.3
		OVO.SVM-R	10.2 ± 0.3	8.8 ± 0.3	7.6 ± 0.3	7.4 ± 0.3	7.2 ± 0.2
		OVA.SVM-L	9.6 ± 0.3	8.3 ± 0.3	7.9 ± 0.3	7.9 ± 0.3	7.9 ± 0.3
		OVA.SVM-R	10.0 ± 0.3	8.7 ± 0.3	8.1 ± 0.3	7.7 ± 0.3	7.2 ± 0.3
		NMC	9.5 ± 0.3	7.6 ± 0.3	6.7 ± 0.2	6.5 ± 0.2	6.1 ± 0.2
		NNC	11.9 ± 0.3	9.3 ± 0.3	7.8 ± 0.2	7.3 ± 0.2	7.4 ± 0.3
	
	ANOVA+Correlation [11]	OVO.SVM-L	6.9 ± 0.3	7.5 ± 0.3	7.4 ± 0.3	7.5 ± 0.3	7.7 ± 0.3
		OVO.SVM-R	7.6 ± 0.3	7.1 ± 0.3	6.4 ± 0.3	6.6 ± 0.2	6.7 ± 0.3
		OVA.SVM-L	7.1 ± 0.3	6.9 ± 0.3	7.1 ± 0.3	8.1 ± 0.3	7.8 ± 0.3
		OVA.SVM-R	7.9 ± 0.3	7.4 ± 0.3	7.1 ± 0.3	7.4 ± 0.3	6.7 ± 0.3
		NMC	7.8 ± 0.3	6.3 ± 0.3	5.7 ± 0.2	5.1 ± 0.2	5.3 ± 0.2
		NNC	9.8 ± 0.3	8.0 ± 0.3	7.5 ± 0.3	7.3 ± 0.3	6.7 ± 0.3

Here *m*-fold corresponds to the case when *m *top most dominant genes are used for each class. For example, the column labeled 3-fold represents the results using 15 genes (3 dominant genes from each of the 5 classes) for both the CNS and Lung Cancer data sets.

In this context it is worth emphasizing that many genes may have discriminating power and hence can be considered marker genes but the dominant and dormant genes are special types of markers and all marker genes are not necessarily dominant/dormant genes. GDI is designed to identify dominant/dormant genes, if present. Moreover, any method of gene selection should be theoretically/conceptually appealing. Use of the OVA strategy may select useful genes for classification but it is not conceptually/theoretically appealing and may lead to potential problems. We have already explained it once and we again reemphasize it here. In the OVA.SNR approach, for a *k*-class problem, to select marker genes, for a class, say class *c*, we divide the data set into two groups, data from class *c *and the pooled data from the remaining *k *- 1 classes. Clearly, the mean and standard deviation of the pooled group do not represent any useful information about the remaining classes. For example, the mean of *k *- 1 pooled classes may fall in a region which may not even have any data points in its neighborhood. Moreover, use of statistics like *t*-statistic makes certain assumptions about the distribution of data in each class. Even if the assumptions are satisfied for each class, it may not (usually will not) be satisfied for the pooled class. The pooling of samples will also affect the ANOVA+Correlation method. The adverse influence of pooling samples from different classes will become more serious if there are several classes. In such a case, the pooled group will be of much higher size than any individual group and hence its influence will also be stronger.

Consequently, this may make the correlation based method fail to recognize overlapped structure between expression levels from different classes. Thus, use of such OVA scheme for gene selection is not conceptually appealing. But this must not be taken to infer that OVA.SNR or ANOVA+Correlation will not be able to select useful genes, nor our intention is to claim that GDI will not select poor genes.

**Figure 17:**
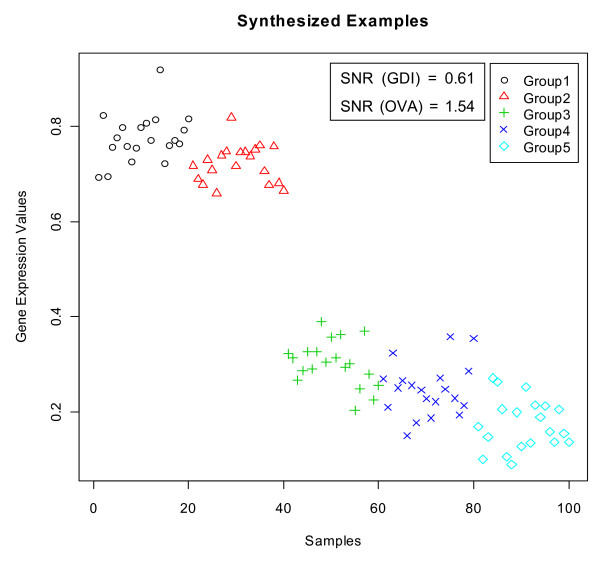
A synthesized example to illustrate a false positive dominant gene identified by SNR (OVA).

We now illustrate with a synthetic data set that SNR (OVA) can lead to false positive dominant/dormant genes. Figure 17 shows the expression values of a five-class data where each class has the same number of samples and roughly the same standard deviation. It is clear that this gene is not a dominant gene. The GDI value for the black class is 0.61 while SNR (OVA) for the same class is 1.54. Note that, since we are using one-versus-all philosophy, a SNR value of 1.54 is expected to be much more significant than a GDI value of 0.61. The most significant difference between SNR (OVA) and GDI methods is that the value of SNR (OVA) is influenced by samples from all other group while GDI uses a comparison of only two selected groups with the highest mean values.

Depending on the data sets the behavior of these three methods (GDI.Dominant, OVA.SNR and ANOVA+Correlation) may be similar in terms of classifier performance, but dominant/dormant genes identified may be different. An important distinctive feature of GDI over OVA is that it can effectively reduce the false positive cases. Even though, the set of top significant dominant/dormant genes from each class identified by these three methods may be similar, the importance (priority) of those genes as dominant/dormant genes may be different.

## Conclusion

We have proposed generalizations of the SNR index for multiclass problems through the introduction of two indices, *GDI*_*Dom *_and *GDI*_*Dor*_. These have led us to define dominant genes and dormant genes with respect to a set of related diseases/cancers. Both dominant and dormant genes have class specific signatures and hence can be used to design useful diagnostic prediction systems. We have explained that good dominant genes are very useful for diagnosis and usually are expected to be present. However, strong dormant genes may not always be available, but if they exist, they are also quite useful for diagnosis. Based on the GDI values we have proposed a mechanism for selecting a set of useful biomarkers that may play significant roles in the biology of the set of diseases (here cancers) under consideration and can be used to design useful diagnostic prediction systems. It is possible to design other methods of discovering biomarkers using the GDIs. Our experimental results suggest that the proposed method can identify good biomarkers.

In order to establish the utility of the dominant and dormant genes we have considered four multi-category cancer data sets. First, we have analyzed the roles of some of the dominant and dormant genes in cancer biology. Then we have used visual assessment techniques to assess the level of dominancy/dormancy of the genes. For these we have used scatterplot of individual gene to assess each gene separately and also have used Sammon's projection to get an idea about the overall quality (discriminating power) of a set of genes selected by our GDIs. These plots have clearly revealed the class specific signatures of the genes selected by the GDIs.

To further demonstrate the utility of the identified genes, we have used six classifiers. Our experiments show that a few dominant genes can yield very good prediction accuracies. We have compared our results with published results and found that the dominant genes identified by our method can result in a comparable performance usually with fewer genes than other methods. But as explained earlier, it may be difficult to find strong dormant genes and hence usually we require more dormant genes than dominant genes to achieve comparable classification performance. When dominant genes are combined with dormant genes, the performance of the system usually, but not necessarily, improves. It would be better to design diagnostic systems using dominant genes and the result of the diagnosis then can be authenticated/validated using the dormant genes, if they exist.

## Materials and methods

### Data sets

#### SRBCT data set [13]

The cDNA microarray data set contains 63 samples from 4 classes of childhood small round blue cell tumors (SRBCT): 23 Ewing sarcomas (EWS), 8 Burkitt lymphomas (BL), 12 neuroblastomas (NB), and 20 rhabdomyosarcomas (RMS). Each sample is represented by 2308 genes. In addition, an independent data set with 6 EWS, 3 BL, 6 NB, and 5 RMS samples are used in the validation process. Both data sets are available at .

#### Leukemia data set [14]

This Affymetrix high-density oligonucleotide array data set contains 57 samples from 3 classes of leukemia: 20 acute lymphoblastic leukemia (ALL), 17 mixed-lineage leukemia (MLL), 20 acute myelogenous leukemia (AML), each with 12582 genes. In addition, an independent data set with 4 ALL, 3 MLL, and 8 AML samples are further used in the validation process. Both data sets are available at .

#### CNS data set [15]

This is also an Affymetrix high-density oligonucleotide microarray data set containing 42 samples from 5 different tumors of the central nervous system (CNS): 10 medulloblastomas (MD), 10 malignant gliomas (MGlio), 10 atypical teratoid/rhabdoid tumors (Rhab), 8 primitive neuro-ectodermal tumors (PNET), and 4 human cerebella tumors (Ncer). For this data set each sample is represented by 7129 genes. This data set is available at .

#### Lung Cancer data set [16]

This Affymetrix high-density oligonucleotide array data set contains 203 samples in 12600 dimensions. There are 5 categories: 139 lung adenocarcinomas (Adeno), 21 squamous cell lung carcinomas (SQ), 20 pulmonary carcinoids (COID), 6 small-cell lung cancer (SCLC), and 17 normal lung specimens (Normal). This data set can be obtained from 

### Preprocessing

For the Leukemia and CNS data sets, in the preprocessing step the gene expression values smaller than 100 are raised to 100; while expression values greater than 16000 are set to 16000, and then the expression values are subjected to a base 10 logarithmic transformation. After that, the distribution of gene expression values in each sample is adjusted to zero mean and unit variance. For the SRBCT data set, we do not make any change to the gene expression values as that had already been preprocessed in the original data source [13]. For these three data sets, we adopt the same data preprocessing protocols as in [8]. For the Lung Cancer data set (not used in [8]), we use the same preprocessed data as used in [16] without doing any additional preprocessing.

### Experiment design

In order to confirm the efficacy of our proposed new gene selection method and to make proper comparisons, we followed the same experimental protocols as used in [8]. First, for gene selection, in addition to our proposed method, we have used two gene selection strategies mentioned in [8]: maximum margin criterion-based recursive feature elimination (MMC-RFE) and support vector machine-based recursive feature elimination (SVM-RFE). Here we have not implemented the MMC-RFE and SVM-RFE algorithms, but simply extracted the results from [8].

Second, for evaluating the performance of each gene selection method, we have used the repeated random splitting methodology utilized in [8] in which the samples (not including the independent test data that are available for the SRBCT and Leukemia) are partitioned randomly into a training set and a test set such that the training and test sets maintain the same proportions of samples from different classes as in the whole data set. The training set consists of two-thirds of the entire sample set, and test set consists of the remaining one-third of the samples. This random training-test splitting is repeated 100 times. For each such random training-test splitting (called outer level splitting), we again randomly split the training set 100 times to produce a smaller training set. In this inner-level splitting, we use three-fourth of the training data for finding dominant and dormant genes, which are then used to evaluate the performance of classifiers on the outer level test data. This classifier performance evaluation process is explained using the following step-algorithm, *Classifier Performance Evaluation.*

#### Algorithm *Classifier Performance Evaluation*

   1. **Outer Loop**: Repeat 100 times – Classifier performance estimation.

      1.1 Partition the data set *X *into *XTR *(training set) and *XTS *(test set), such that *XTR *= $\frac{r}{s}$*X*, *XTS *= *X *- *XTR*, *r *<*s*; for example, here we use *r *= 2, *s *= 3, *XTR *= $\frac{2}{3}$*X.*

      1.2 **Inner Loop**: Repeat 100 times – Frequency-based gene selection.

         1.2.1 Partition the training set *XTR *into *XTR*_1 _and *XTR*_2_, such that *XTR*_1 _= $\frac{p}{q}$*XTR*, *XTR*_2 _= *XTR *- *XTR*_1_, *p *<*q*; here we use *p *= 3, *q *= 4, *XTR*_1 _= $\frac{3}{4}$*XTR*.

         1.2.2 Use *XTR*_1 _to compute GDIs for each gene and then note the set of best *m *dominant and *m *dormant genes for each class. Update the frequency of the selected genes.

      1.3 Generate the set *SG *with the *m *most frequently occurring dominant and dormant genes from each class.

      1.4 Train classifier(s), *C*, using *XTR *considering all or part of the genes in *SG*.

      1.5 Evaluate classifier(s), *C*, on the test set *XTS*.

   2. Classifier evaluation: Summarize performance of the classifiers over the 100 outer level trials.

In our investigation in Step 1.2.2 and Step 1.3 we have used *m *= 5. In Step 1.4 we have used six kinds of classifiers for comparison (three of them are used in [8]): the Nearest Mean Classifier, the Nearest Neighbor Classifier, and four kinds of the Support Vector Machine Classifiers. The adopted SVM classifiers include the one-versus-one SVM with linear kernel (OVO.SVM-L), the one-versus-one SVM with Gaussian kernel (also called SVM with Radial Basis Function, OVO.SVM-R), the one-versus-all SVM with linear kernel (OVA.SVM-L), and the one-versus-all SVM with Gaussian kernel (OVA.SVM-R). Note that, only the SVM.OVA-L was used in [8]. We have implemented the NMC and NNC classifiers; while for application of SVM to multi-class problems, we have used the e1071 library of R  which is based on the LIBSVM . For SVMs, the training data are further randomly split into two equal parts (training and validation) for determining the optimal hyper-parameters for the SVM classifiers. The optimal hyper-parameters are then used to design SVM classifiers with the training data and their performance is evaluated on the test data. Here for *C *(the constant for regularization), we use four choices {1, 10, 100, 1000} and for the spread of Gaussian kernel *γ*, we consider eight choices {0.0001, 0.001, 0.01, 0.1, 1, 10, 100, 1000}.

### Gene dominant and dormant indices (GDI)

As we mentioned in Background, our main contribution is to develop a gene evaluation index, called "Gene Dominant/Dormant Index (GDI)", to select significant genes for multicategory classification problems. This GDI concept is similar in spirit to the Signal-to-Noise ratio (SNR), broadly adopted for gene selection in two-class problems [2], but the GDI can be applied to multicategory problems. Moreover, GDI further helps to identify dominant and dormant genes as defined next.

#### Dominant Gene

A gene that is over-expressed in only one of the classes and under-expressed in the remaining classes. Thus a dominant gene is defined with respect to a set of diseases/classes and it has a very strong class specific signature.

#### Dormant Gene

A gene that is under-expressed in only one of the classes but over-expressed in the remaining classes. Thus a dormant gene is also defined with respect to a set of diseases/classes and it also has a strong class specific signature.

**Figure 18:**
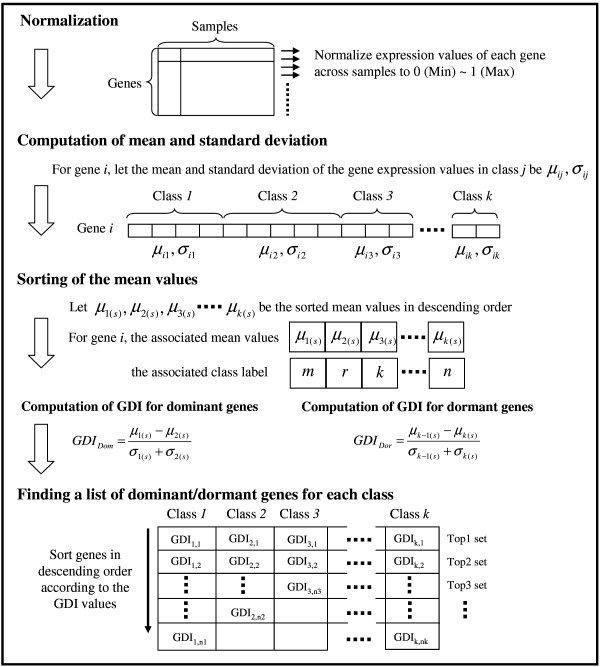
Computation of GDI and finding the lists of dominant genes and dormant genes.

From the above definitions, it is clear that dominant genes, if any, will be good biomarkers because such genes are expected to play active roles for the disease. It also appears that finding a dominant gene may not be a difficult task, particularly for a given set of cancers, because usually some genes will be highly expressed for a particular type of cancer. But dormant genes may not always be available in a given set of diseases as the requirements of dormant genes are harder to satisfy. It is easy to visualize that both dormant genes and dominant genes will have high discriminating power. Moreover, one can design a diagnostic system using the dominant genes and then can authenticate the decisions using information available with the dormant genes. These can lead to more reliable diagnostic systems. In simulation results we demonstrate that we can make more accurate prediction for several multiclass problems based on dominant or dormant genes selected by the GDI criterion (compared to two existing gene selection methods for multiple classes, such as SVM-RFE [8] and MMC-RFE [8]). For an easy understanding, Fig. 18 depicts the steps involved in the computation of GDI, which are explained next.

#### Normalization

The expression values of each gene are normalized in the range from 0 to 1 across samples. This step preserves the richness in the original expression values for each gene among the samples and helps us to easily visualize the distribution of expression values for the dominant or dormant genes.

#### Computation of mean and standard deviation

For each gene, the mean and standard deviation of the gene expression values in each class are calculated. Let the mean and standard deviation for gene *i *in class *j *be *μ*_*ij*_, *σ*_*ij*_.

#### Sorting of the mean values

For notational simplicity, to explain the computation of the GDI for gene *i*, we ignore the index *i*. We sort *μ*_*j*_; *j *= 1, ..., *k *in *descending *order. Let the sorted mean values be *μ*_*j*(*s*)_; *j *= 1, ..., *k*. Suppose *μ*_1(*s*) _is the mean for class *m*. This means that the gene under consideration is most highly expressed in class *m*. Similarly, if *μ*_2(*s*) _corresponds to class *r*, then if we exclude class *m*, then amongst the remaining classes this gene has the highest expression level on average in class *r*. Thus, if the gene under consideration has a distinct class specific signature, then *μ*_1(*s*) _and *μ*_2(*s*) _must be well separated and if that is not so, then this gene cannot be a dominant gene. Note that, to make this conclusion, we do not need to look at the mean values corresponding to other classes. We can do so because we have sorted the class means in descending order.

#### Computation of GDI for *dominant *genes

Now we define the *GDI*_*Dom *_for the gene under consideration as:

(1) $$GDI_{Dom}=\frac{\mu_{1(s)}-\mu_{2(s)}}{\sigma_{1(s)}+\sigma_{2(s)}}$$

As discussed above, the index at Equation 1 can be computed for each gene and then the *GDI*_*Dom *_values can be sorted in descending order. A higher value of *GDI*_*Dom *_indicates that the gene for the *m*-th class is significantly over-expressed compared to the *r*-th class and obviously it is more strongly over-expressed compared to the remaining classes. Thus, it is a dominant gene for class *m *or 1(*s*). Dominant genes, if exist, will appear at the top of the sorted list. A set of genes can then be selected from this sorted list for further processing. Note that, for a two class problem, although we do not use the absolute value in the numerator, because of the sorting, Equation 1 is exactly the same as that of Golub's SNR index [2]. In other words, the *GDI*_*Dom *_can be viewed as true generalization of Golub's SNR for a multiclass problem.

#### Computation of GDI for *dormant *genes

However, the *GDI*_*Dom *_in Equation 1 will not be able to find the dormant genes, if any. In order to find the dormant genes we can proceed as follows. If the gene under consideration is a dormant one, then it will be unexpressed for one class but at least moderately expressed for all of the remaining classes. In this case, (*μ*_*k*-1(*s*) _- *μ*_*k*(*s*)_) should be considerably high, where *μ*_*k*(*s*) _is the last value in the sorted sequence; in other words, it is the mean expression level for the class in which the gene under consideration is least expressed. Thus, we define the *GDI*_*Dor *_for identifying the dormant gene as

(2) $$GDI_{Dor}=\frac{\mu_{k-1(s)}-\mu_{k(s)}}{\sigma_{k-1(s)}+\sigma_{k(s)}}$$

Note that, Equation 1 uses the class mean values and standard deviations of the top two classes in the sorted list while Equation 2 uses the class means and standard deviations corresponding to the last two values in the sorted list. Consequently, if *GDI*_*Dor *_is significantly high for a gene, then this gene is a dormant gene for the class represented by *k*(*s*).

It is easy to see that for a two class problem, *GDI*_*Dor *_reduces to the SNR of [2]. Thus both *GDI*_*Dom *_and *GDI*_*Dor *_can be viewed as generalizations of SNR. We can combine Equations 1 and 2 and write in a convenient manner as in Equation 3.

(3) $$GDI_{x}=\frac{\left|\mu_{p}-\mu_{q}\right|}{\sigma_{p}+\sigma_{q}}$$

In Equation 3 when *x *= *Dom*, *p *and *q *correspond to the top two classes, respectively, in the sorted list and when *x *= *Dor*, then *p *and *q *correspond to the last two classes in the sorted list, respectively.

We want to emphasize that a dominant gene is dominant for a class with respect to the given set of *classes/groups *under consideration. For example, given the SRBCT group, a gene may be dominant for the Neuroblastoma class implying that this gene is highly expressed for the Neuroblastoma cases but unexpressed for the other three types of childhood cancers. Now if we augment the set of four childhood cancers by one more type, then this particular gene may not remain dominant with respect to the group of five childhood cancers. Similar is the case with dormant genes.

#### Finding a list of dominant/dormant genes for each class

After calculating the *GDI*_*Dom *_values of all genes, a list of dominant genes for *each class *can be obtained as follows. For each gene, the *GDI*_*Dom *_is associated to the class represented by 1(*s*); in other words, it is associated to the class corresponding to the top element in the sorted list. In this way, every gene is associated with a class and a value of dominancy as expressed by *GDI*_*Dom*_. We can now sort the genes associated with a particular class according to the *GDI*_*Dom *_values. In this way we get a sorted list for each class. We can now select useful genes for a class from the top of the list. Clearly, when selecting the dominant genes, the higher the *GDI*_*Dom*_, the more dominant the gene is. A similar procedure can be applied for the generation of a list of dormant genes for each class using the *GDI*_*Dor *_values.

### Gene selection strategy

If we use several dominant (or dormant or both kinds of) genes from each class ranked according to *GDI*_*Dom *_values to design diagnostic systems, we are expected to get sufficient discriminating power for all classes in multi-class discrimination problems. But since in each resampling experiment we may get a different set of dominant (dormant) genes for a class, it would be better to aggregate the output of several resampling experiments. Different strategies are possible for this. Next we propose one such strategy:

#### Frequency-based method

The gene selection scheme is displayed in Algorithm *Gene Selection*. It proceeds as follows. In each of the 100 trials, we select the top *m *(= 5) dominant (dormant) genes for each class to compute the frequency with which each such gene appears as a candidate gene for a class. A good dominant (dormant) gene is likely to appear more frequently. In order to find the set of interesting (marker) genes for each class we select the top five most frequently occurring genes. However, some class may have more than five genes with strong class specific signatures. If that happens, we should include those genes also if our goal is to find the set of interesting (marker) genes, not just designing of a classifier. Hence, in addition to the top five genes, if there are other genes with frequency of appearance 50 or more (in 100 trials) we also consider those genes important. In this manner we find a set of genes that may be biologically interesting. But all these genes may not be necessary for designing a classifier, because for a *k*-class discrimination, even a set of less than *k *good genes may be adequate. Tables 1, 2, 3, 4 are generated by this scheme.

#### Algorithm *Gene Selection*

   1. Repeat 100 times.

      1.1 Partition the data set *X *into *XTR *and *XTS*, such that *XTR *= $\frac{p}{q}$*X*, *XTS *= *X *- *XTR*, *p *<*q*; here we use *p *= 2, *q *= 3, *XTR *= $\frac{2}{3}$*X*.

      1.2 Use *XTR *to compute GDIs for each gene.

      1.3 Find the set of best *m *dominant and *m *dormant genes for each class.

      1.4 Note the frequency of the selected genes.

   2. Generate the set of dominant (dormant) genes with the *m *most frequently occurring dominant (dormant) genes from each class.

### Permutation test to assess statistical significance of GDI indices

To assess the statistical significance of the GDI indices associated with the identified dominant and dormant genes, a permutation test has been performed. The procedure followed is summarized below. Both un-adjusted *p*-values and *q*-values adjusted for multiple comparisons are computed. Let *G *be the total number of genes and *S *be the total number of sample points.

**(1) **Given an expression matrix *D *(*x*_*gs *_is the expression intensity of gene *g *and sample unit *s*; 1 ≤ *g *≤ *G*, 1 ≤ *s *≤ *S*) with class labels (*y*_*s*_, 1 ≤ *s *≤ *S*), we compute the gene dominant index *GDI*_*Dom*_, *m*_*g *_and gene dormant index *GDI*_*Dor*_, *r*_*g*_, for each gene *g*.

**(2) **Randomly permute the class labels *y*_*s *_for *B *times. In the *b*th permutation (1 ≤ *b *≤ *B*), compute $m_{g}^{\left(b\right)}$, the new *GDI*_*Dom *_and $r_{g}^{\left(b\right)}$, the new *GDI*_*Dor *_for gene *g *using the expression matrix *D *and the permuted labels $y_{s}^{\left(b\right)}$.

**(3) **The *p*-value of the observed dominant GDI, *m*_*g*_, for gene *g *is

(4) $$p(m_{g})=\frac{\sum_{b=1}^{B}\sum_{g^{\prime }=1}^{G}I(m_{g^{\prime }}^{\left(b\right)}\geq m_{g})}{G\times B},$$

where *I*(·) is an indicator function that takes the value one when true and zero otherwise. Similarly the *p*-value of the observed dormant GDI, *r*_*g*_, is

(5) $$p(r_{g})=\frac{\sum_{b=1}^{B}\sum_{g^{\prime }=1}^{G}I(r_{g^{\prime }}^{\left(b\right)}\geq r_{g})}{G\times B}.$$

**(4) **To account for the multiple tests being performed in the *G *genes, *q*-values of the observed *m*_*g*_and *r*_*g *_are calculated as

(6) $$\begin{array}{ccc} q(m_{g})=\frac{\sum_{b=1}^{B}\sum_{g^{\prime }=1}^{G}I(m_{g^{\prime }}^{\left(b\right)}\geq m_{g})}{\sum_{g^{\prime }=1}^{G}I(m_{g^{\prime }}\geq m_{g})\times B} & \text{and} & q(r_{g})=\frac{\sum_{b=1}^{B}\sum_{g^{\prime }=1}^{G}I(r_{g^{\prime }}^{\left(b\right)}\geq r_{g})}{\sum_{g^{\prime }=1}^{G}I(r_{g^{\prime }}\geq r_{g})\times B}. \end{array}$$

## Authors' contributions

All authors contributed significantly to the investigation. YST, CTL, IFC, and NRP together formulated the new indices. YST and IFC implemented the algorithms and conducted the experiments. GCT designed and carried out the statistical experiment. IFC and NRP led and coordinated the investigation. CTL, IFC, and NRP wrote the manuscript. All authors have read and approved the final manuscript.
